# Comparative Transcriptomics Reveals Important Genes Underlying Heat-Tolerant Sterility in Photo-Thermo-Sensitive Male Sterile Wheat in Seed Production Environments

**DOI:** 10.3390/biom16030368

**Published:** 2026-02-28

**Authors:** Jieru Yue, Shaohua Yuan, Qiling Hou, Xiaocong Hao, Zhijie Ye, Jinsai Chen, Fengting Zhang, Changping Zhao, Zihan Liu, Hui Sun

**Affiliations:** Beijing Key Laboratory of Crop Molecular Design and Intelligent Breeding, Beijing Key Laboratory of Molecular Genetics in Hybrid Wheat, Institute of Hybrid Wheat, Beijing Academy of Agriculture and Forestry Sciences, Beijing 100097, China; yuejieru@baafs.net.cn (J.Y.); yuanshaohua@baafs.net.cn (S.Y.); houqiling@baafs.net.cn (Q.H.); haoxiaocong@baafs.net.cn (X.H.); yezhijie@baafs.net.cn (Z.Y.); chenjinsai@baafs.net.cn (J.C.); zhangfengting@baafs.net.cn (F.Z.); zhaochangping@baafs.net.cn (C.Z.)

**Keywords:** wheat PTMS lines, heat, RNA-sequencing, WGCNA, safe seed production

## Abstract

Maintaining stable male sterility is fundamental for ensuring the genetic purity and productivity of two-line hybrid wheat. However, unexpected heat events during the fertility-sensitive period can induce fertility restoration in photo-thermo-sensitive male sterile (PTMS) lines, posing a major threat to hybrid seed production. In this study, we identified two BS-type PTMS lines, BS166 and BS192, that consistently maintained sterility under heat stress in a seed-production environment, indicating strong heat-tolerant sterility. To uncover the molecular basis underlying this stability, we compared four BS-type PTMS lines exhibiting contrasting heat responses through field assessments, controlled heat treatments, transcriptome sequencing, and weighted gene co-expression network analysis (WGCNA). A total of 19,105 differentially expressed genes were identified, with the bisque4 module showing a significant correlation with seed setting rate. KEGG enrichment analysis revealed that starch and sucrose metabolism, cutin, suberin, and wax biosynthesis, fatty acid biosynthesis, and plant hormone signal transduction pathways were highly associated with heat-tolerant sterility. Core genes within these pathways displayed transcriptional stability in BS166 and BS192 but were strongly induced in heat-sensitive lines. In situ hybridization and RT-qPCR further confirmed tapetum-specific expression of *TaBGLU32* and *TaLACS1*. Based on these findings, we propose a regulatory model explaining how PTMS lines maintain sterility stability under heat stress.

## 1. Introduction

Exploiting heterosis in wheat (*Triticum aestivum* L.) is a useful approach for increasing the yield of wheat per-unit area. This can aid in meeting the demands of a growing global population, ensuring food security, and mitigating the impacts of increasingly frequent extreme environmental conditions [1]. The two-line hybrid wheat system, with photo-thermo-sensitive male sterile (PTMS) lines at its core, which has been independently developed in China, represents a major breakthrough in hybrid wheat breeding both domestically and internationally. Currently, China’s hybrid wheat industry has entered the primary stage of large-scale production and application. However, instability in hybrid seed yield and quality has become a major restriction on its development [2,3]. In hybrid production, male sterility is the most effective mechanism for exploiting heterosis in self-pollinated crops [4]. Therefore, cultivating PTMS lines of wheat for safe and stable hybrid seed production is key to improving seed production efficiency and stability [5].

Wheat PTMS lines were first discovered by Sasakuma in 1979 [6]. Since the 1990s, diverse types of wheat PTMS lines have also been found in China, including the ES and C49S spring-type PTMS germplasm [7,8], the BS type PTMS germplasm [9], as well as newly identified thermo-sensitive or photo-thermo sensitive materials, such as LT-1-3A [10], YS [11], BNS [12,13], and 337S [14]. Owing to their strong male sterility and restoration ability, and the ease of selecting superior dominant traits, they have become a major research model in male-sterility studies [15]. It is well-documented that the fertility of PTMS lines is regulated by photoperiod and temperature conditions [16]. Based on photoperiod-temperature control technology, short-day low temperature can directionally induce male sterility and achieve large-scale hybrid seed production. Long-day high temperature reversibly restores fertility, enabling the self-propagation of sterile lines [16]. This reversible fertility conversion has substantial value for hybrid seed production and sterile line propagation [16].

Previous studies have shown that at sufficiently low temperatures following spike differentiation, the pollen viability of sterile lines rapidly shifts from fertile to completely sterile [3,17,18]. Furthermore, variations exist in the fertility-sensitive periods among different types of sterile lines, with BS-type lines exhibiting sensitivity from the connective to the uninucleate stage [19]. The fertility changes of BS20 are mainly caused by temperature, with a critical temperature point of 10–12 °C [19]. Below the critical temperature, BS20 remains sterile and is not affected by photoperiod [19]. Above it, fertility increases with rising temperature, and within 12–14 °C, fertility is further enhanced by longer photoperiods [19]. This unique fertility pattern restricts the stability of sterile-line propagation and hybrid seed production [20]. Particularly in recent years, abnormal heat events have frequently occurred during the fertility-sensitive period of sterile lines, resulting in numerous failures in two-line hybrid wheat seed production [20]. A similar phenomenon has been reported in rice, although the fertility conversion conditions in rice sterile lines are opposite to those in wheat [20]. Rice sterile lines require long-day and high-temperature conditions for hybrid seed production. Low-temperature stress during the fertility-sensitive period of rice sterile lines in seed production areas significantly reduces seed purity and leads to seed production failure [21,22,23,24]. At present, there are few reports on the effects of heat conditions on wheat sterile lines in seed production areas.

Currently, numerous studies have investigated the molecular mechanisms underlying male sterility, including premature tapetal degradation leading to nutrient deficiency in developing pollen, which eventually results in sterility [25]. Alterations in actin, soluble proteins, soluble sugars, proline, RNA [26], NAD^+^ kinase and NADP phosphatase activities, antioxidant enzyme activities [27,28], Ca^2+^ concentration and distribution, and plant hormones have all been implicated in the failure of wheat sterile lines to produce viable pollen [29]. In addition, abnormal mitochondrial DNA structures and RNA editing may also be associated with male sterility [30,31]. However, the molecular basis of heat-responsive sterility maintenance in wheat sterile lines remains unclear.

The discovery of male sterile wheat has greatly advanced the exploitation of heterosis. To date, studies on male sterile lines have been conducted in various crops, such as rapeseed, rice, and cotton [14]. To elucidate the molecular basis underlying heat-tolerant sterility in seed production areas, BS-type PTMS lines were used as experimental materials in this study. Heat stress conditions were simulated, and the fertility performance of the sterile lines was evaluated. Transcriptome sequencing was employed to identify heat-tolerant sterility-related differentially expressed genes (DEGs). Furthermore, weighted gene co-expression network analysis (WGCNA) was applied to identify key genes that may regulate sterility stability under heat stress, followed by preliminary functional analysis. This research lays a foundation for investigating the regulatory basis of heat-tolerant sterility in PTMS lines in seed production environments and fills an important knowledge gap in this field.

## 2. Materials and Methods

### 2.1. Plant Materials, Experimental Design, and Sampling

The experimental materials consisted of BS-type PTMS lines of wheat, including BS166 (BS366 × Taishan 23), BS192 (BS366 × Jingdong 18), BS294 (05GA1183 (Zu 2 × Jimai 36) × Lunxuan 987) and BS1453 (CP56 × Beinong 2). They exhibit male sterile under short-day and low-temperature conditions (Dengzhou, Henan; 34°40′ N, 112°21′ E), while fertility under long-day and high-temperature conditions (Shunyi, Beijing; 40°08′ N, 116°39′ E), and their fertility conversion sensitive period spanned the connective to uninucleate stage. All BS-type PTMS lines were developed by the Institute of Hybrid Wheat, Beijing Academy of Agriculture and Forestry Sciences. They were sown in flower pots (26 cm × 23 cm), with 20 pots per genotype, and grown in the field of Shunyi, Beijing. Amounts of 0.88 g of diamine phosphate and 0.63 g of urea were applied as base fertilizers. At the stage when the seedlings had developed four leaves, an additional topdressing of 0.63 g was applied. After vernalization, all plants were transferred to a greenhouse for subsequent cultivation. At the connective stage, plants were transferred to an artificial climate chamber for heat and control treatment in 2024 and 2025. The normal sterile condition was set to 12 h at 12 °C as the control (CK); the heat treatment (H) consisted of 12 h at 23 °C; the 12 h period corresponded to the photoperiod, and temperatures represented daily mean values. The daily temperature difference between treatments was 10 °C. During the experiment, the humidity level was maintained at 70%, ensuring a stable moisture content in the environment, while light intensity was maintained at 125 µ Mol/(m^2^·s) (Table S1). The sterile lines were exposed to heat treatment for 3 days and then moved to the climate chamber at 12 h 12 °C to grow alongside the control materials. After the end of the booting stage, they were all moved to the greenhouse for normal growth until maturity. Anthers of the heat-treated and control sterile lines during the fertility-sensitive period were collected, respectively, with three biological replicates per genotype per treatment. Samples were stored in a −80 °C refrigerator for transcriptome sequencing and subsequent analysis.

### 2.2. Statistics of Maximum Temperature Variation in the Seed Production Area and Fertility Assessment of the Sterile Lines

Dengzhou, Henan Province, served as the seed production area. The fertility sensitive period of the sterile lines is generally from 15 March to 5 April. Accordingly, we retrieved the daily maximum temperature data of Dengzhou from 15 March to 5 April over the past three years (2023–2025) through a weather website (www.tianqi.com (accessed on 10 October 2025)). We also calculated the seed setting rates of the 99 sterile lines planted each year. The formula for the calculation of the seed setting rate was as follows: Seed setting rate = (Number of grains per spike/(Number of spikelets × 2)) × 100% [32].

### 2.3. Phenotypic Characterization

The florets of sterile lines in the trinucleate stage were sampled, and their phenotypic characteristics were photographed using a Leica S9i microscope (Leica Microsystems, Wetzlar, Germany). Pollen viability was assessed using I_2_-KI staining [33], and images were captured with a Leica DM500 microscope (Leica Microsystems, Wetzlar, Germany). Additionally, ten uniformly growing main stem spikes per pot were covered with self-pollination bags prior to anthesis, with three pots evaluated per genotype per treatment. Seed-setting rates were subsequently calculated. Adobe Illustrator CS6 (v16.0.0) was employed to assemble the phenotypic images of the sterile lines’ florets, and Graphpad Prism 8.0.2 was used to draw the seed setting rate and pollen iodine staining ratio plots and to conduct significance analyses.

### 2.4. Bioinformatic Analysis of RNA-Seq Data

Anthers from BS166, BS192, BS294, and BS1453 during the fertility-sensitive period under different treatments were used for RNA-Sequencing (RNA-Seq), encompassing a total of 24 samples. The transcriptome sequencing service was provided by Beijing igeneCode Biotech Co., Ltd. (Beijing, China). RNA extraction and library construction followed the procedures described by Niu [32]. Sequencing was performed on the Illumina HiSeq4000 (Illumina, Inc., San Diego, CA, USA) sequencing platform. The sequencing adapters and primer sequences in the reads were removed, and the low-quality data in the raw data obtained from sequencing were filtered to obtain clean data for subsequent analysis. Clean reads were aligned to the wheat reference genome (IWGSC RefSeq v1.0) using HISAT v2.2.1 (Hierarchical Indexing for Spliced Alignment of Transcripts). Gene expression levels were quantified as FPKM (Fragments Per Kilobase of transcript per Million base pairs sequenced), a widely used parameter that accounts for sequencing depth and transcript length.

### 2.5. Identification, Hierarchical Cluster Analysis, and Functional Annotation of DEGs

The analysis of DEGs was conducted using the DESeq2 v1.34.0 R package, and genes with |log2(FoldChange)| > 1 and adjusted *p*-value < 0.05 were selected as DEGs. Hierarchical clustering analysis of DEGs in each group was performed using the pheatmap v1.0.13 R package to explore gene expression patterns. To further clarify the functional annotation of DEGs, GO and KEGG pathway analyses were conducted, using the “phyper” function of R v4.1.2, with a corrected *p*-value < 0.05 considered significant for both.

### 2.6. Weighted Gene Co-Expression Network Analysis of DEGs

WGCNA is a system biology method used to analyze gene expression data, designed to identify gene modules and explore their relationships with phenotypes. By clustering modules and phenotypic data, the core module associated with the target trait was identified. In this study, 19,105 DEGs and seed setting rate data served as inputs to conduct WGCNA using the WGCNA v1.70.3 R package. The pickSoftThreshold function was used to determine the optimal soft-thresholding power, which was set to 12, with other parameters set to default values. The proximity relationship was calculated based on the soft threshold, and the topological overlap matrix (TOM) was constructed. Gene modules were then identified through hierarchical clustering. Module–trait correlations and corresponding *p*-values were calculated to identify the key module where the heat-tolerance genes for sterility were placed. Finally, subsequent functional analysis was conducted on these genes.

### 2.7. Gene Expression and Real-Time Quantitative Reverse Transcription PCR (RT-qPCR) Analysis

The expression patterns of key DEGs were analyzed and visualized as a heatmap using TBtools v2.323. Total RNA was extracted from the anthers of four sterile lines under both heat treatment and control conditions. For *TaLACS1* and *TaBGLU32* expression analysis in roots, stems, and leaves, total RNA was also extracted from these tissues. RT-qPCR analysis was performed after reverse transcription. The RNA extraction, reverse transcription, and RT-qPCR methods were performed following the methods of Luo et al. (2022) [34]. The *TaActin* was used as a reference gene. RT-qPCR primers were designed using the online website NCBI-Primer BLAST (https://www.ncbi.nlm.nih.gov/tools/primer-blast/ (accessed on 5 November 2025)), with primer sequences listed in Table S2. RT-qPCR data were analyzed by two-way analysis of variance (two-way ANOVA).

### 2.8. In Situ Hybridization Analysis

In situ hybridization was performed on wheat anthers. Anthers were fixed in in situ hybridization fixative solution (Servicebio, Wuhan, Hubei, China, Cat. No. G1113), dehydrated, embedded in paraffin, and sectioned into 4 μm slices [35]. Specific digoxigenin (DIG)-labeled sense and antisense RNA probes targeting *TaLACS1* and *TaBGLU32* were synthesized in vitro [36]. Paraffin sections were deparaffinized, rehydrated, treated with proteinase K, post-fixed, and acetylated prior to pre-hybridization. Hybridization was conducted overnight at 42 °C using denatured DIG-labeled antisense probes, with sense probes serving as negative controls [37]. After post-hybridization washes to remove unbound probes, immunodetection was carried out using HRP-conjugated anti-DIG antibody. Hybridization signals were visualized using the DAB Substrate Kit (Servicebio, Wuhan, Hubei, China, Cat. No. G1212). Each section was counterstained with hematoxylin (Servicebio, Wuhan, Hubei, China, Cat. No. G1004), dehydrated through an ethanol series, cleared in xylene, and mounted with neutral balsam (Servicebio, Wuhan, Hubei, China, Cat. No. G1409). Images were captured using a Nikon Eclipse CI microscope (Nikon Instruments Inc., Melville, NY, USA) equipped with a Nikon DS-U3 imaging system (Nikon Instruments Inc., Melville, NY, USA).

## 3. Results

### 3.1. Relationship Between Heat Conditions in the Seed Production Area and the Fertility of Sterile Lines

**Figure 1:**
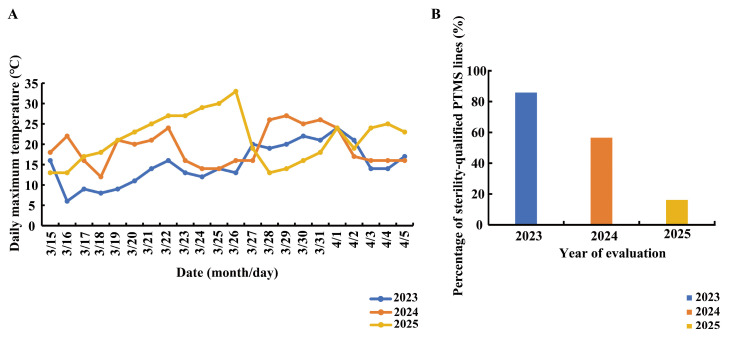
Daily maximum temperature variation during the fertility-sensitive period and fertility assessment of sterile lines in the seed production area from 2023 to 2025. (**A**) Variation in daily maximum temperatures during the fertility-sensitive period of sterile lines in the seed production area from 2023 to 2025. (**B**) The percentage of sterility-qualified PTMS lines in the seed production area from 2023 to 2025.

The results of the fertility evaluation test indicated that the percentage of sterile lines with the seed setting rate below 1%—the threshold defining qualified sterility-ranged widely from 16.16% to 85.86% across the three years. Pronounced interannual differences in the fertility of sterile lines existed, with the highest percentages recorded in 2023 (85.86%), followed by 2024 (56.57%), whereas a sharp decline occurred in 2025, when only 16.16% of lines met the sterility standard (Figure 1B). These results indicate substantial year-to-year fluctuations in fertility performance under field conditions, suggesting a strong influence of environmental factors. Given the well-established temperature sensitivity of BS-type PTMS lines during the fertility-sensitive period, we further analyzed detailed meteorological data from 2023 to 2025, emphasizing temperature dynamics between 15 March and 5 April, which corresponds to the critical fertility-determining stage of the sterile lines. Distinct differences in maximum temperature profiles were observed among the three years. The recorded daily maximum temperatures during this period were 24 °C in 2023, 27 °C in 2024, and 33 °C in 2025. Notably, in 2025, two consecutive days exceeded 30 °C, and an additional day surpassed 28 °C. In contrast, maximum temperatures in both 2023 and 2024 never exceeded 28 °C throughout the same developmental window.

Moreover, from 20 March to 26 March in 2025, daily maximum temperatures were consistently higher than those recorded during the corresponding periods in 2023 and 2024 (Figure 1A), indicating a short-term but pronounced heat event during the fertility-sensitive stage. Previous studies and production experience indicate that the optimal average temperature for maintaining sterility in BS-type PTMS lines is 12 °C, with an upper tolerance threshold of 17 °C. Therefore, the temperature conditions in 2025 substantially exceeded the favorable range for stable sterility maintenance. Collectively, these results demonstrate a clear association between abnormal heat events in the seed production area and a sharp reduction in the proportion of sterile lines meeting the sterility standard. Based on the temperature profiles observed during fertility instability, an average temperature of 23 °C with a daily maximum of 28 °C was selected as a suitable heat stress regime for subsequent controlled experiments, aiming to simulate field-relevant thermal perturbations without imposing extreme lethality.

### 3.2. Effects of Heat Treatment on the Seed Setting Rate of Sterile Lines

**Figure 2:**
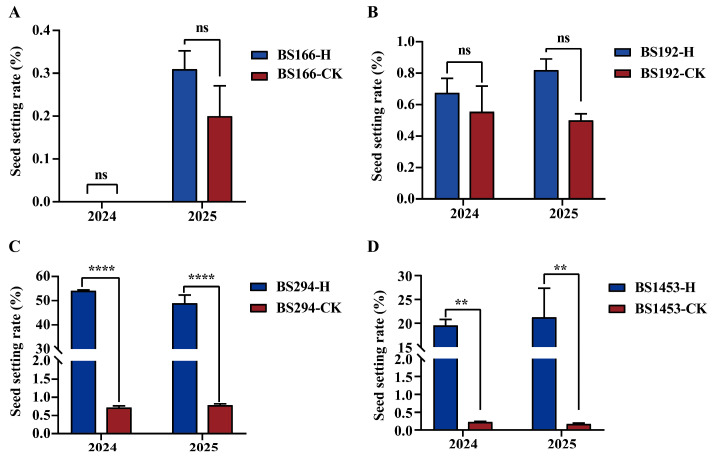
Seed setting rate analysis of sterile lines under control and heat-treatment conditions in 2024 and 2025 (**A**–**D**). **** *p* < 0.0001, ** *p* < 0.01, ns: not significant.

Controlled environment experiments showed that for BS166, seed-setting rates under heat treatment and control conditions ranged from 0–0.31% and 0–0.2%, respectively, in 2024 and 2025. For BS192, the seed setting rates were 0.68–0.82% under heat treatment and 0.5–0.55% in the control group in 2024 and 2025. There was no significant difference in the seed setting rates between the heat treatment and the control for either BS166 or BS192. Notably, the seed setting rates of BS166 and BS192 under different treatments were all below 1%, indicating stable male sterility (Figure 2A,B). By contrast, heat treatment significantly enhanced the seed setting rates of BS1453 and BS294. For BS294, the seed setting rates increased to 48.97–54.07% under heat treatment, significantly higher than the values of the control group (0.72–0.79%). Similarly, BS1453 exhibited an increase in seed setting rates to 19.56–21.27% after heat treatment, showing a highly significant difference compared with the control group (0.18–0.23%) (Figure 2C,D). The results indicated that the seed setting rates of the four sterile lines were less than 1% under the control condition, consistently exhibiting male sterility. However, only BS166 and BS192 retained sterility under heat stress, indicating strong heat-tolerant sterility.

### 3.3. Phenotypic Characteristics of Sterile Lines Under Different Treatments

**Figure 3:**
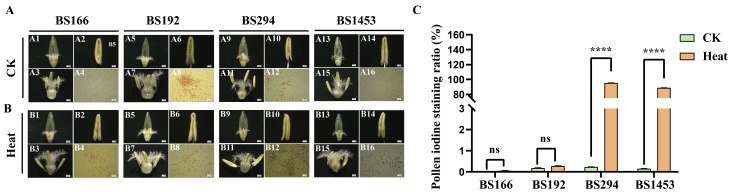
Phenotypic characteristics and pollen iodine staining ratios of sterile lines under different treatments. The phenotypes of dissected florets (**A1**,**A5**,**A9**,**A13**), the reproductive organs (**A3**,**A7**,**A11**,**A15**), anthers (**A2**,**A6**,**A10**,**A14**) and pollen staining (**A4**,**A8**,**A12**,**A16**) of sterile lines under CK condition. (**B**) The phenotypes of dissected florets (**B1**,**B5**,**B9**,**B13**), the reproductive organs (**B3**,**B7**,**B11**,**B15**), anthers (**B2**,**B6**,**B10**,**B14**) and pollen staining (**B4**,**B8**,**B12**,**B16**) of sterile lines under heat treatment. (**C**) Iodine-stained pollen ratios of sterile lines under heat and CK conditions. Scale bars: 1 mm in (**A**,**B**). **** *p* < 0.0001, ns: not significant.

Microscopic examination of anther morphology and pollen viability revealed distinct phenotypic responses among the four sterile lines. Under the control condition, pollen from all lines exhibited a yellowish-brown color after I_2_-KI staining, with pollen iodine staining ratios ranging from 0% to 0.23%. This indicated that their pollen had basically no viability, resulting in male sterility (Figure 3(A4,A8,A12,A16,C)). After heat treatment, the pollen of BS294 and BS1453 were mostly stained blue, with pollen iodine staining ratios of 95.28% and 88.74%, respectively, demonstrating high pollen viability and fertility restoration (Figure 3(B12,B16,C)). In contrast, BS166 and BS192 showed no obvious staining after heat treatment, with pollen iodine staining ratios of only 0.05% and 0.28%, respectively, thereby maintaining male sterility (Figure 3(B4,B8,C)). Additionally, the anthers of BS166 and BS192 were relatively small and shrunken under both control and heat stress conditions. Regarding BS294 and BS1453, their anthers appeared smaller under control conditions; however, they were larger and plumper after heat treatment (Figure 3A,B). Thus, BS166 and BS192 exhibited considerable heat-tolerant sterility, maintaining stable male sterility even under elevated temperatures.

### 3.4. Overview of RNA-Seq Data Analysis

RNA-Seq analysis was performed using anther samples from each sterile line at the fertility-sensitive period under control conditions (BS166-CK, BS192-CK, BS294-CK, BS1453-CK) and heat treatment conditions (BS166-H, BS192-H, BS294-H, BS1453-H), with three biological replicates per sample per treatment. Following quality control, the RNA-seq analysis yielded an average of 10.77 Gb of clean data. The Q20 and Q30 scores exceeded 97.14% and 91.89%, respectively. The GC content ranged from 51.97% to 54.14%. Alignment of the clean reads of each sample with the reference genome (IWGSC RefSeq v1.0) resulted in total mapped reads and unique mapped reads percentages that varied from 93.83 to 97.63% and 85.60 to 92.79%, respectively (Table S3).

### 3.5. Identification and Functional Analysis of DEGs

**Figure 4:**
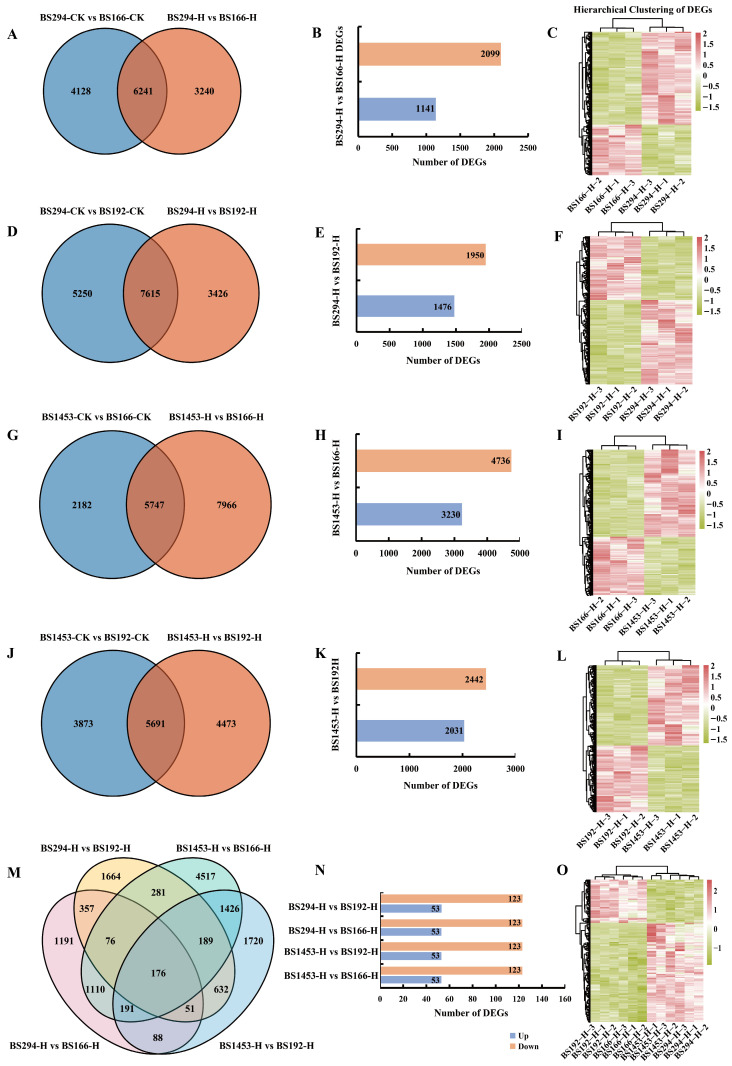
Identification and analysis of DEGs associated with heat-tolerant sterility in sterile lines. (**A**,**D**,**G**,**J**,**M**): Venn diagrams of DEGs in sterile lines under different treatments. (**B**,**E**,**H**,**K**,**N**): Bar charts depicting numbers of upregulated and downregulated DEGs associated with heat-tolerant sterility in sterile lines under heat treatment. (**C**,**F**,**I**,**L**,**O**): Hierarchical clustering heatmaps of DEG expression profiles in sterile lines under heat treatment.

To probe into the molecular basis underlying heat-tolerant sterility in the seed production area, we identified DEGs associated with differential fertility responses under heat treatment. Pairwise comparisons were performed between sterile lines that exhibited different fertility levels after heat treatment: BS294-H vs. BS166-H, BS294-H vs. BS192-H, BS1453-H vs. BS166-H, and BS1453-H vs. BS192-H. Corresponding control comparisons (BS294-CK vs. BS166-CK, BS294-CK vs. BS192-CK, BS1453-CK vs. BS166-CK, BS1453-CK vs. BS192-CK) were conducted to eliminate inherent background differences among genotypes. Across all comparisons, 9481, 10,369, 11,041, 12,865, 13,713, 7929, 10,164, and 9564 DEGs were identified in BS294-H vs. BS166-H, BS294-CK vs. BS166-CK, BS294-H vs. BS192-H, BS294-CK vs. BS192-CK, BS1453-H vs. BS166-H, BS1453-CK vs. BS166-CK, BS1453-H vs. BS192-H, and BS1453-CK vs. BS192-CK, respectively (Figure S1). After removing background differences, a total of 19,105 unique DEGs associated with heat-tolerant sterility were retained. Among these, 3240, 3426, 7966, and 4473 DEGs were identified in BS294-H vs. BS166-H, BS294-H vs. BS192-H, BS1453-H vs. BS166-H, and BS1453-H vs. BS192-H, respectively (Figure 4A,D,G,J, Tables S4–S7). A total of 1141 DEGs were up-regulated, and 2099 were down-regulated in BS294-H vs. BS166-H, 1476 DEGs were up-regulated, and 1950 were down-regulated in BS294-H vs. BS192-H, 3230 DEGs were up-regulated, and 4736 were down-regulated in BS1453-H vs. BS166-H, 2031 DEGs were up-regulated, and 2442 were down-regulated in BS1453-H vs. BS192-H (Figure 4B,E,H,K, Tables S4–S7). Additionally, 176 coincident DEGs were identified across all four comparisons, including 53 consistently upregulated and 123 consistently downregulated genes (Figure 4M,N, Tables S4–S7). In summary, the number of down-regulated DEGs was consistently higher than that of up-regulated DEGs in all comparisons of sterile lines under the heat treatment.

Clustering analysis of DEG expression levels within each comparison, as well as clustering of the 176 coincident DEGs, consistently showed that most genes were down-regulated in BS166-H and BS192-H, whereas they were up-regulated in BS294-H and BS1453-H (Figure 4C,F,I,L,O). These results suggest that most of these DEGs contribute to sterility maintenance. Subsequently, gene ontology (GO) and kyoto encyclopedia of genes and genomes (KEGG) pathways analysis were performed on the 176 coincident DEGs. The results revealed significant enrichment in biological processes, such as “defense response”, “tyrosine biosynthetic process”, “multidimensional cell growth”, “aromatic amino acid family biosynthetic process, prephenate”, and “response to biotic stimulus” (Figure S2A). Enriched KEGG pathways included “MAPK signaling pathway-plant”, “Phenylalanine, tyrosine and tryptophan biosynthesis”, “Plant hormone signal transduction”, “Glycine, serine and threonine metabolism”, “Glycosphingolipid biosynthesis-ganglio series”, “Glyoxylate and dicarboxylate metabolism”, “Galactose metabolism”, and “Flavonoid biosynthesis” (Figure S2B).

### 3.6. WGCNA of DEGs

**Figure 5:**
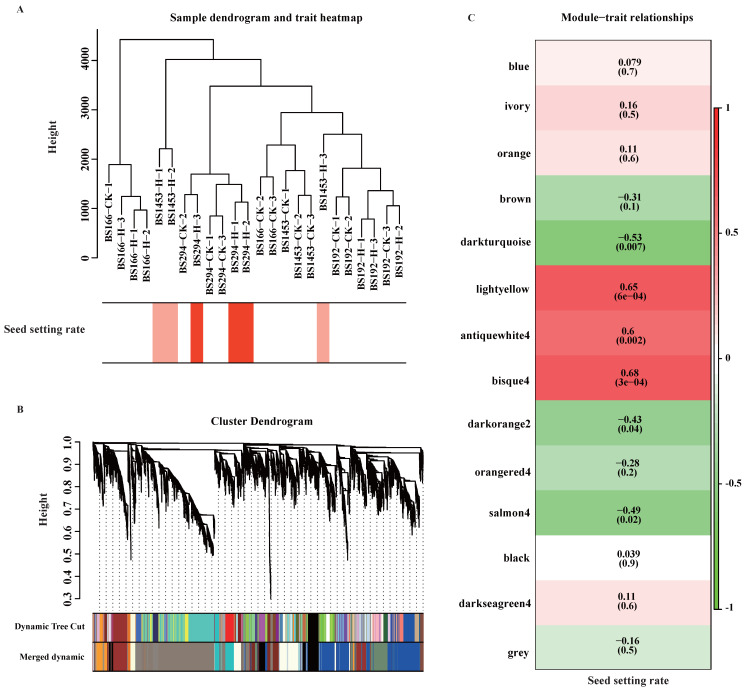
Weighted gene co-expression network analysis of DEGs. (**A**) Sample clustering and heatmap of seed setting rates. (**B**) Cluster dendrogram and module identification. (**C**) Analysis of the module-trait relationships. Note: In the heatmap of (**A**), darker colors correspond to higher seed setting rates.

To further identify key genes involved in sterility maintenance under heat stress, WGCNA was performed using seed-setting rate data and the 19,105 DEGs. Firstly, a sample clustering tree and a trait heatmap were constructed. The results showed that the clustering distance between BS166-CK and BS166-H was relatively short, as did BS192-CK and BS192-H. This indicates that the overall gene expression profiles of BS166 and BS192 did not change significantly before and after the heat treatment. However, the clustering distance between BS1453-CK and BS1453-H was large, reflecting substantial transcriptional reprogramming under heat stress. These results were consistent with the phenotypic data. Unexpectedly, the clustering distance between BS294-CK and BS294-H was relatively short (Figure 5A), which initially appeared inconsistent with the dramatic phenotypic shift from sterility to high fertility (seed setting rate: 0.72–0.79% vs. 48.97–54.07%, Figure 2C). However, this pattern supports the hypothesis that BS294 represents a “fragile equilibrium” state. Its transition from sterility to fertility may require only modest transcriptional changes, suggesting that BS294 is positioned near a critical regulatory threshold under control conditions. While many genes are already transcriptionally elevated under control conditions (potentially reflecting a compromised baseline regulatory state), crossing the fertility threshold may require only modest additional changes in a subset of key regulatory genes under heat stress. This contrasts sharply with BS1453, where substantial transcriptional reprogramming is required for fertility restoration (as evidenced by the highly distinct clustering between BS1453-CK and BS1453-H). The observed differences in clustering patterns represent distinct regulatory states: BS166 and BS192 possess robust homeostatic mechanisms that counteract heat-induced transcriptional changes; BS1453 demonstrates major transcriptional activation under heat, while BS294 maintains an intrinsically unstable state where minimal perturbations are sufficient to initiate fertility restoration. The trait heatmap revealed that BS294-H displayed the highest seed setting rate, followed by BS1453-H, whereas BS166 and BS192 had the lowest seed setting rate. Samples with similar expression profiles and seed setting rates were clustered together, indicating a strong correlation between gene expression patterns and the trait (Figure 5A).

**Figure 6:**
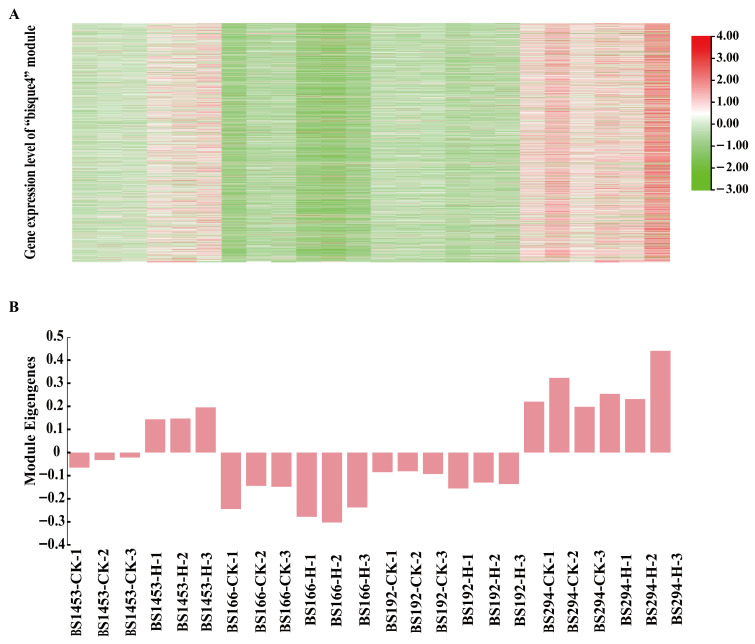
Gene expression patterns of the bisque4 module across treatments and samples. (**A**) Heatmap of gene expression levels in the bisque4 module across treatments and samples. (**B**) Bar plot of the MEbisque4 (Module Eigengene of the bisque4 module) values, illustrating its expression dynamics across treatments and samples.

Subsequently, the gene co-expression modules were divided through dynamic tree pruning and module merging strategies, which yielded 14 independent co-expression modules (Figure 5B). Module-trait correlation analysis identified the “bisque4” module as the core module linked to seed setting rate (r = 0.68, *p* = 3 × 10^−4^), suggesting that genes within this module may participate in the regulation of heat-tolerance of male-sterile lines (Figure 5C). Further analysis of gene expression and module eigengenes (MEs) of “bisque4” revealed that BS166, BS192, and BS1453 exhibited relatively lower baseline expression of many bisque4 genes under control conditions, whereas BS294 exhibited elevated expression in control conditions even before heat treatment (Figure 6). Despite this elevated baseline expression, BS294 still maintained male sterility under control conditions (seed setting rate 0.72–0.79%, Figure 2C), suggesting that high expression of fertility-related genes alone is insufficient to trigger fertility restoration. However, BS294 failed to maintain sterility under heat stress (seed setting rate 48.97–54.07%), indicating that its transcriptional regulatory network, despite being capable of maintaining sterility under optimal conditions, lacks the robustness to buffer against environmental high-temperature perturbations. These findings suggest that the maintenance of heat-tolerant sterility in PTMS wheat is determined by the capacity to maintain transcriptional stability under stress, rather than absolute expression levels at a single timepoint. Based on these findings, BS166 and BS192 exhibited stable male sterility under heat stress, and genes in the “bisque4” module might be the key to heat-tolerant sterility.

### 3.7. Functional Analysis of Key DEGs Related to Heat-Tolerant Sterility

**Figure 7:**
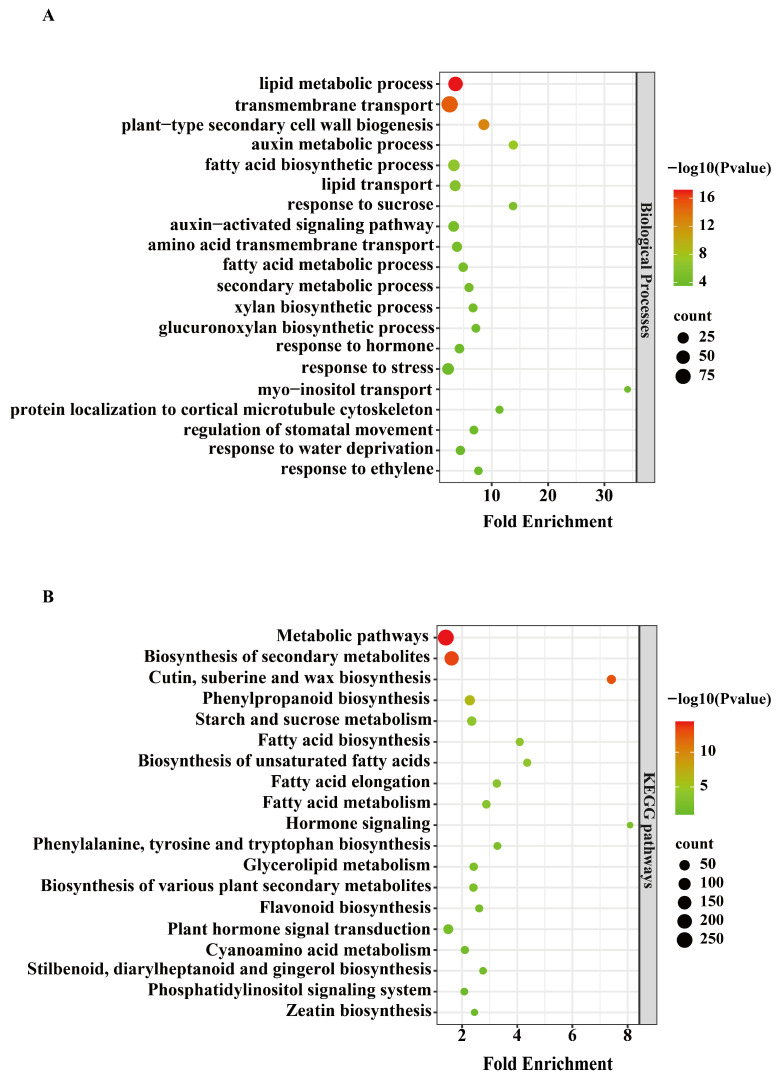
Functional enrichment analysis of genes in the bisque4 module. (**A**) GO biological process enrichment analysis of genes in the bisque4 module. (**B**) KEGG pathway enrichment analysis of genes in the bisque4 module.

To elucidate the meaning of the transcriptional response underlying the heat tolerance of PTMS lines under heat stress, GO and KEGG pathways enrichment analyses were performed on genes in the “bisque4” module. GO biological process enrichment revealed significant overrepresentation of terms related to “lipid metabolic process”, “transmembrane transport”, “auxin metabolic process”, and “fatty acid biosynthetic process”. Among these, “lipid metabolism process” exhibited the highest fold enrichment and statistical significance (−log_10_P), and also contained the largest number of genes. Additionally, processes related to sucrose response, hormone regulation, and stress response were also significantly enriched (Figure 7A), which indicated that the functions of key genes possibly related to sterility regulation under heat stress were either involved in basic metabolic regulation or abiotic stress response.

The KEGG pathways enrichment results further revealed that these genes were predominantly involved in metabolic pathways, including “Fatty acid metabolism”, “Phenylpropanoid biosynthesis”, “Cutin, suberin and wax biosynthesis”, “Starch and sucrose metabolism”, “Phenylalanine, tyrosine and tryptophan biosynthesis”, and “Plant hormone signal transduction” pathways (Figure 7B). Integrating these results with known effects of heat on male sterility suggests that the enriched lipid metabolism, secondary metabolism, and stress response-related pathways may participate in sterility maintenance under heat stress by regulating pollen wall structure (such as wax/keratin synthesis and fatty acid metabolism), hormone signal balance (such as auxin metabolism), and energy metabolism (such as fatty acid metabolism). This provides a mechanistic framework for understanding the functional roles of key sterility regulatory genes in heat stress response.

### 3.8. Expression Analysis and RT-qPCR Validation of Genes in Key KEGG Pathways

Further analyses of the expression levels of representative genes from the enriched KEGG pathways were conducted, including long chain acyl-CoA synthetase 1-like (TraesCS1A02G075000) involved in “Fatty acid biosynthesis”, stearoyl-[acyl-carrier-protein] 9-desaturase 5, chloroplast-like (TraesCS5D02G131500 and TraesCS5D02G548700) involved in “Fatty acid metabolism”, very-long-chain aldehyde decarbonylase GL1-4-like (TraesCS2D02G381900) and peroxygenase 1-like (TraesCS2D02G382300) involved in “Cutin, suberine and wax biosynthesis”, auxin-responsive protein IAA20-like (TraesCS7B02G029100) involved in “Plant hormone signal transduction”, beta-glucosidase 32-like (TraesCS5D02G303000) involved in “Starch and sucrose metabolism”, alpha-galactosidase-like (TraesCS5B02G170600), involved in “Glycerolipid metabolism”, peroxidase 43-like (TraesCS4D02G102600) involved in “Phenylpropanoid biosynthesis” (Figure 7B), and the differential expression analysis data for these genes are shown in Tables S4–S7.

**Figure 8:**
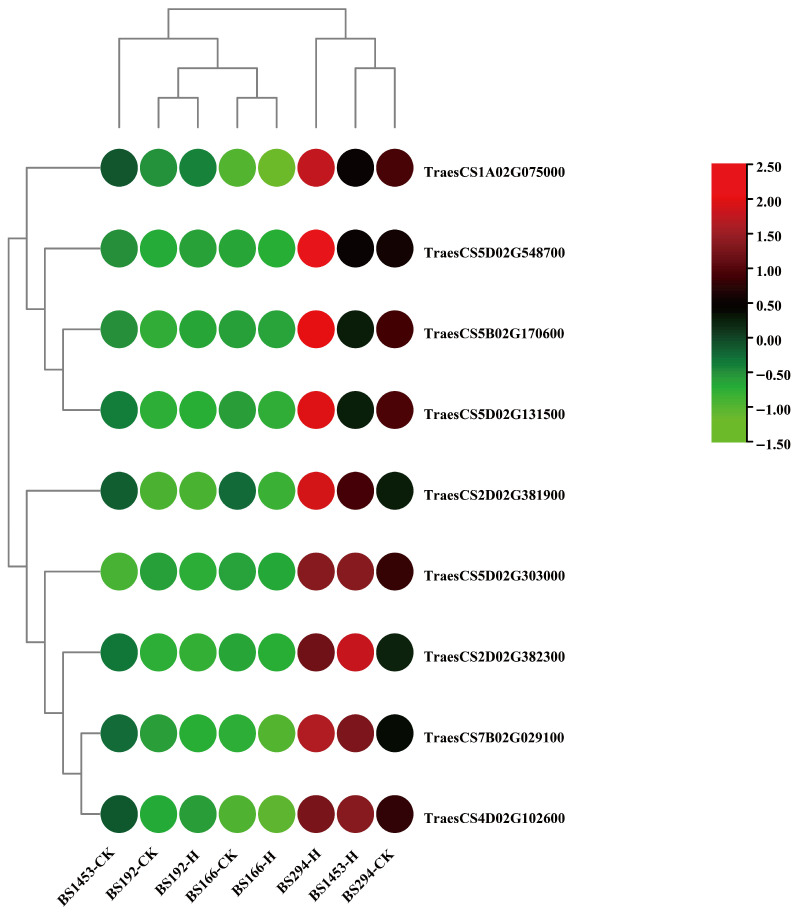
Expression analysis of core genes in key KEGG pathways.

**Figure 9:**
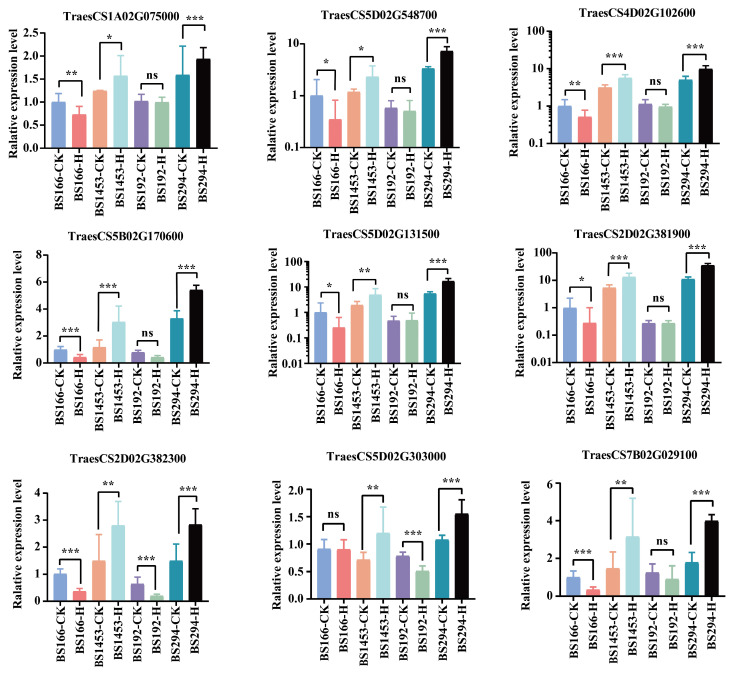
RT-qPCR validation of the expression pattern of core genes associated with heat-tolerant sterility. Relative expression levels were calculated by the 2^−ΔΔCt^ method, with three biological replicates and technical replicates. *** *p* < 0.001, ** *p* < 0.01, * *p* < 0.05, ns: not significant.

Transcriptome data showed that these genes exhibited markedly higher expression in BS294-H, BS1453-H, and BS294-CK. However, the expression levels of the genes were consistently higher in BS294-H and BS1453-H than in their respective controls, whereas they remained low in both the control and heat-treated samples of BS166 and BS192 (Figure 8). RT-qPCR validation confirmed these patterns: the expression levels of nine core genes related to four important KEGG pathways were significantly decreased or showed no significant difference in BS166 and BS192, while they were significantly increased in BS1453 and BS294, after heat treatment (Figure 9). These findings were fully consistent with the trends observed in the transcriptome data, which further demonstrated the high accuracy and reliability of the RNA-seq transcriptome data. Meanwhile, these genes may be involved in maintaining sterility under heat stress. Collectively, these results reinforce the hypothesis that heat-tolerant sterility depends on the capacity to maintain suppressed expression of fertility-promoting genes under heat stress, rather than on particular absolute expression levels. The observation that BS294 already exhibits relatively high baseline expression of these genes (Figure 8 and Figure 9) yet remains sterile under control conditions further underscores that transcriptional stability under stress, not baseline expression per se, is the critical determinant of sterility maintenance.

### 3.9. Spatiotemporal Expression Patterns of TaLACS1 and TaBGLU32

**Figure 10:**
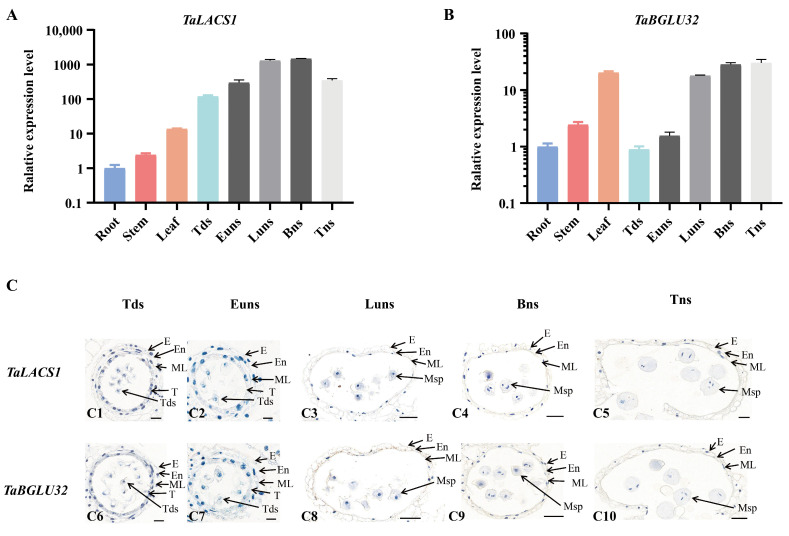
Analysis of the spatiotemporal expression patterns of *TaLACS1* and *TaBGLU32*. (**A**,**B**) RT-qPCR analysis of *TaLACS1* and *TaBGLU32* expression in different tissues (roots, stems, leaves) and different anther developmental stages. (**C**) In situ hybridization analysis of *TaLACS1* and *TaBGLU32* expression across anther developmental stages. Tds, tetrad stage; Euns, early uninucleate stage; Luns, late uninucleate stage; Bns, binucleate stage; Tns, trinucleate stage; E, epidermis; En, endothecium; ML, middle layer; T, tapetum; Msp, microspores. Scale bars: 25 μm (**C1**,**C5**,**C6**); 20 μm (**C2**,**C7**); 50 μm (**C3**,**C4**,**C8**,**C9**,**C10**).

*TaLACS1* (TraesCS1A02G075000; long chain acyl-CoA synthetase 1-like) and *TaBGLU32* (TraesCS5D02G303000; beta-glucosidase 32) were selected for further analysis due to their pronounced downregulation in BS166 and BS192 under heat stress conditions (Figure 9), together with their strong functional relevance to pollen development [38,39,40]. To probe into the functional relevance of wheat male sterility, we first characterized its expression profiles across diverse tissues and during different stages of anther development. Tissue-specific expression analysis revealed that *TaLACS1* was minimally expressed in roots, stems, and leaves, whereas its expression levels were robustly elevated in anthers, highlighting a striking anther-preferential expression pattern. In contrast, *TaBGLU32* exhibited substantial expression in leaves, as well as high expression in anthers, suggesting roles in both vegetative growth and reproductive development. Notably, during anther development, *TaBGLU32* expression displayed a progressive upward trend, remaining highly expressed from the tetrad stage through the early uninucleate, late uninucleate, binucleate, and trinucleate stages, exhibiting the highest expression during the binucleate and trinucleate stages. By comparison, *TaLACS1* expression increased from the tetrad to binucleate stages, with the highest expression during the late uninucleate and binucleate stages, followed by a decline at the trinucleate stage (Figure 10A,B). RNA in situ hybridization further confirmed the tissue-specific localization of the genes. Strong hybridization signals for both *TaLACS1* and *TaBGLU32* were detected specifically in the tapetum of the anther wall and in developing microspores, with the most intense signals observed during the tetrad and early uninucleate stages (Figure 10C). Collectively, these findings indicate that *TaLACS1* and *TaBGLU32* possibly exert regulatory roles in pollen fertility, and their tightly regulated spatiotemporal expression patterns may be strongly associated with the manifestation of male sterility in wheat.

## 4. Discussion

### 4.1. Heat Stress as a Critical Threat to the Stability of PTMS-Based Hybrid Wheat Seed Production

Photo-thermo-sensitive male sterile lines constitute the core germplasm for two-line hybrid wheat breeding systems. Their fertility is reversibly modulated by photoperiod and temperature cues, obviating the requirement for maintainer and restorer lines and thereby markedly simplifying hybrid seed production. This system represents a pivotal technological advance toward the global commercialization of hybrid wheat [2,3]. Nevertheless, widespread adoption of PTMS lines remains severely limited by the instability of male sterility under fluctuating environmental conditions, particularly extreme temperature events [2].

Extensive research has established that elevated temperatures impair pollen viability, disrupt pollination, exacerbate the excessive accumulation of reactive oxygen species (ROS), and ultimately result in reduced seed setting rate in common wheat [41]. Importantly, as PTMS lines of wheat exhibit fertility under long-day and high-temperature conditions but sterility under short-day and low-temperature conditions, the impact of heat stress on PTMS lines may be different from that on common wheat.

In this study, we conducted a multi-year analysis of seed-setting rates in BS-type PTMS lines under field seed production conditions and correlated these phenotypic outcomes with maximum temperature fluctuations during the fertility-sensitive stage. Our findings revealed that years featuring episodic extreme heat events were associated with aberrant increases in seed-setting rates among sterile lines, resulting in compromised sterility and posing a direct threat to hybrid seed production safety. Controlled heat stress experiments further confirmed that among the four BS-type PTMS lines examined, BS1453 and BS294 exhibited rapid fertility restoration under heat stress, whereas BS166 and BS192 maintained robust and stable sterility. These results demonstrate that although extreme heat broadly disrupts sterility regulation, heat-tolerant sterile genotypes do exist. This phenotypic divergence provided a robust biological foundation for dissecting the molecular mechanisms underlying heat-tolerant sterility maintenance, rather than sterility per se.

### 4.2. Heat-Tolerant Sterility Is Determined by Network Stability Rather than Static Gene Expression Levels

Temperature-dependent fertility regulation in wheat constitutes a complex, multi-layered regulatory process that extends beyond single-gene effects [33]. Previous studies have demonstrated that temperature influences fertility primarily through regulating tapetum function, pollen wall formation, and carbohydrate and lipid metabolism during microsporogenesis [42]. For instance, in the wheat *DCL5* double mutant, low temperature induces aberrant tapetum development, defective pollen wall synthesis, and anther indehiscence, whereas heat treatment restores tapetal structure and pollen viability, potentially through early activation of the *PHAS*–24 nt phasiRNA pathway [42]. Similarly, genes such as *WTMS1*, *SCULP1*, and other lipid metabolism-related factors integrate light signaling, reactive oxygen homeostasis, and metabolic flux to modulate pollen development in a temperature-dependent manner [43,44].

Notably, these studies collectively suggest that fertility determination is governed by coordinated gene networks, rather than by the absolute expression levels of individual genes. In this framework, our study addresses a fundamentally distinct biological question from traditional male sterility research: rather than identifying “which genes cause sterility?”, we investigate “which molecular mechanisms confer stability of sterility under heat stress?” This conceptual shift is essential for the accurate interpretation of our transcriptomic results.

All four BS-type PTMS lines examined in this work exhibit complete male sterility under standard sterility-inducing conditions, yet display marked divergence in heat-stress responses. Our data clearly demonstrate that the bisque4 gene lacks a consistent simple linear relationship between expression level and sterility status across genotypes. In particular, BS294 displays relatively high baseline expression of many bisque4 genes under control conditions while remaining phenotypically sterile, a pattern that contradicts the simplistic assumption that “low expression equals sterility.” These observations indicate that static expression levels under a single environmental regime are insufficient to explain sterility outcomes.

Instead, our results demonstrate that the defining feature distinguishing heat-tolerant (BS166 and BS192) from heat-sensitive (BS1453 and BS294) lines is the stability of the transcriptional regulatory network in response to heat perturbation. In BS166 and BS192, bisque4 module genes display minimal transcriptional fluctuations between control and heat treatments, reflecting a robust and buffered regulatory state. In contrast, BS1453 and BS294 exhibit pronounced heat-induced upregulation of this module, coincident with fertility restoration. Therefore, the stability of transcriptional regulation of bisque4-associated genes, rather than simple changes in expression levels, is the core determinant of whether the male-sterile lines can maintain their sterile state under heat stress.

### 4.3. A Two-Tiered Lock Model for Stable Male Sterility Under Heat Stress

Based on the integration of phenotypic, transcriptomic, and pathway-level evidence, we propose a “Two-Tiered Lock” model to explain heat-tolerant sterility in wheat PTMS lines. The first-tier lock comprises baseline transcriptional suppression of fertility-related pathways under sterility-inducing conditions. This layer operates effectively in BS166, BS192, and BS1453, but appears partially compromised in BS294, which exhibits elevated baseline expression of bisque4 genes. Nevertheless, the persistence of near-complete sterility in BS294 under control conditions (with seed-setting rates below 1%) demonstrates that basal transcription level alone is insufficient to determine the sterility trait, but it serves as an important background characteristic of the sterile state. The second-tier lock, which we propose as the decisive determinant of heat tolerance, entails the capacity to preserve this repressed transcriptional state under environmental stress. BS166 and BS192 possess a strong homeostatic buffering capacity: core bisque4 genes, including *BGLU32*, *GL1-4*, *LACS1*, and *IAA20*, remain transcriptionally stable under heat stress. By contrast, BS1453 and BS294 lack this buffering mechanism, leading to coordinated activation of metabolic and hormone signaling pathways and consequent fertility restoration.

The behavior of BS294 is especially instructive, representing a fragile equilibrium state: although its first-tier lock is weakened, sterility is still sustained under optimal conditions, possibly through compensatory action of other genetic factors. However, the absence of a robust second-tier lock renders the system vulnerable to even moderate heat-induced perturbations, destabilizing the network and triggering fertility restoration. This interpretation is supported by the close transcriptional clustering of BS294 under control and heat conditions despite the pronounced phenotypic divergence.

**Figure 11:**
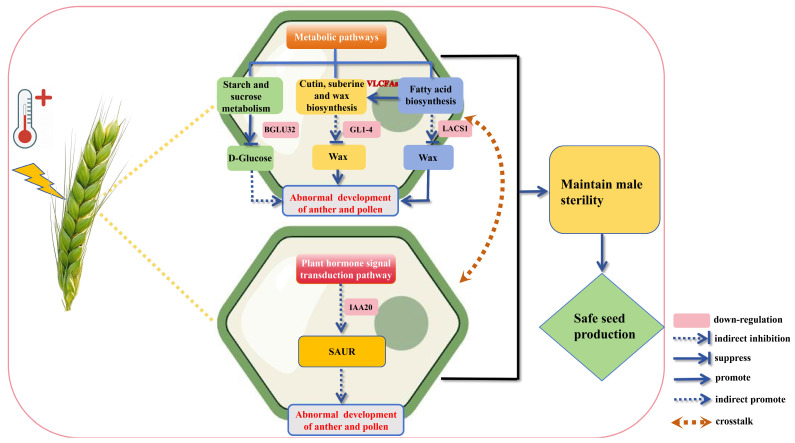
A hypothetical model of heat-tolerant sterility in wheat PTMS lines. Abbreviations: BGLU32 (TraesCS5D02G303000), beta-glucosidase 32-like; GL1-4 (TraesCS2D02G381900), very-long-chain aldehyde decarbonylase-like Glossy1-4; LACS1 (TraesCS1A02G075000), long chain acyl-CoA synthetase 1-like; IAA20 (TraesCS7B02G029100), auxin-responsive protein IAA20-like; SAUR, Small Auxin-Up RNA; VLCFAs, Very-Long-Chain Fatty Acids.

Functionally, the core bisque4 genes are enriched in pathways related to carbohydrate metabolism, cutin and wax biosynthesis, fatty acid metabolism, and auxin signaling. Genes such as *BGLU32*, *GL1-4*, *LACS1*, and *IAA20* have well-established roles in pollen wall formation, tapetal lipid metabolism, and hormone-regulated anther development across plant species [38,39,40,45,46,47,48]. Our in situ hybridization results further confirmed tapetum-specific expression of *TaBGLU32* and *TaLACS1.* Literature reports link male sterility to premature tapetal programmed cell death (PCD), in systems such as sunflower PET-CMS [49], rice HL-CMS [50], and TAZ1-silenced plants [51]. In addition, *AtLACS1* is expressed in microspores and tapetum, and double mutants of *LACS1* and *LACS4* in *Arabidopsis* exhibit blocked pollen-stigma hydration and conditional male sterility [52]. *OsBGLU38* is highly expressed in the cytoplasm and intine of pollen grains from the binucleate stage to the mature pollen stage in rice, with loss-of-function causing intine synthesis defects and complete male sterility [53]. Therefore, we speculate that *TaLACS1* and *TaBGLU32* possess potential neofunctionalization in wheat reproductive tissues. Importantly, these pathways are tightly interconnected: VLCFAs-dependent cutin and wax biosynthesis relies on upstream fatty acid metabolism, while auxin signaling modulates carbohydrate allocation and anther dehiscence. Together, they likely constitute a “metabolism–hormone” bidirectional regulatory network, which acts as the molecular basis of the second-tier lock maintaining sterility under heat stress (Figure 11).

Collectively, our study demonstrates that the bisque4 module genes function as a key regulatory hub for buffering environmental perturbations, rather than as universal determinants for the initial establishment of male sterility. Specifically, we established that these genes reinforce transcriptional stability and environmental robustness, which are critical for maintaining the male-sterile state in PTMS lines under heat stress. This finding provides a conceptual framework for breeding heat-resilient sterile lines to ensure the reliability and scalability of hybrid wheat seed production.

## 5. Conclusions

This study demonstrated that elevated temperatures induce fertility restoration in PTMS wheat lines, thereby compromising sterility under heat stress. Notably, the BS166 and BS192 exhibited strong heat-tolerant sterility, maintaining male sterility. Further using WGCNA, we identified that a set of genes statistically associated with heat-tolerant sterility, which were predominantly enriched in “Starch and sucrose metabolism”, “Cutin, suberin and wax biosynthesis”, “Fatty acid biosynthesis”, and “Plant hormone signal transduction” pathways. Critically, core genes in these pathways displayed constitutively suppressed and stable expression in heat-tolerant lines (BS166 and BS192) under both control and heat stress conditions, but showed significant heat-induced upregulation in heat-sensitive lines (BS1453 and BS294). This differential pattern, i.e., transcriptional stability versus heat-responsive activation, provides strong correlative evidence that the capacity to maintain stable gene expression patterns under heat stress, rather than achieving specific absolute expression thresholds, is a key molecular determinant of heat-tolerant sterility. In situ hybridization and RT-qPCR analyses further revealed that *TaLACS1* and *TaBGLU32* were highly expressed during anther development, indicating that fatty acid biosynthesis and sucrose metabolism may be crucial for maintaining sterility in wheat PTMS lines under heat stress. Finally, we established a hypothetical mechanistic model depicting the molecular pathways through which genes regulate sterility in response to heat stress. This study provides a theoretical foundation for ensuring safe hybrid seed production using male sterile lines under heat and offers practical guidance for identifying and breeding wheat PTMS lines with stable sterility.

## Data Availability

The raw sequence data reported in this paper have been deposited in the Genome Sequence Archive (Genomics, Proteomics & Bioinformatics 2025) in the National Genomics Data Center (Nucleic Acids Res 2025), China National Center for Bioinformation/Beijing Institute of Genomics, Chinese Academy of Sciences (GSA: CRA035543), which are publicly accessible at https://ngdc.cncb.ac.cn/gsa (accessed on 19 December 2025).

## Supplementary Materials

The following supporting information can be downloaded at: https://www.mdpi.com/article/10.3390/biom16030368/s1. Figure S1: Statistics of all DEGs in each library; Figure S2: Functional enrichment analysis of 176 coincident DEGs. (A) GO biological process enrichment analysis of 176 coincident DEGs. (B) KEGG pathways enrichment analysis of 176 coincident DEGs; Table S1: Heat and control conditions for the sterile lines; Table S2: Primer sequences for RT-qPCR; Table S3: Summary statistics of RNA-seq data from sterile lines under control and heat treatment conditions; Table S4: Unique DEGs in BS294-H vs. BS166-H; Table S5: Unique DEGs in BS294-H vs. BS192-H; Table S6: Unique DEGs in BS1453-H vs. BS166-H; Table S7: Unique DEGs in BS1453-H vs. BS192-H.

## Abbreviations

The following abbreviations are used in this manuscript:

DEGs	Differentially Expressed Genes
GO	Gene Ontology
KEGG	Kyoto Encyclopedia of Genes and Genomes
WGCNA	Weighted Gene Co-expression Network Analysis
RT-qPCR	Real-Time quantitative reverse transcription PCR
PTMS	Photo-Thermo-sensitive Male Sterility
RNA Seq	RNA-Sequencing
