# Supplementary figures and images for: Comparative Transcriptomics Reveals Important Genes Underlying Heat-Tolerant Sterility in Photo-Thermo-Sensitive Male Sterile Wheat in Seed Production Environments
Source: Biomolecules. 2026 Feb 28;16(3):368. doi: 10.3390/biom16030368 (PMC13024202; doi:10.3390/biom16030368)

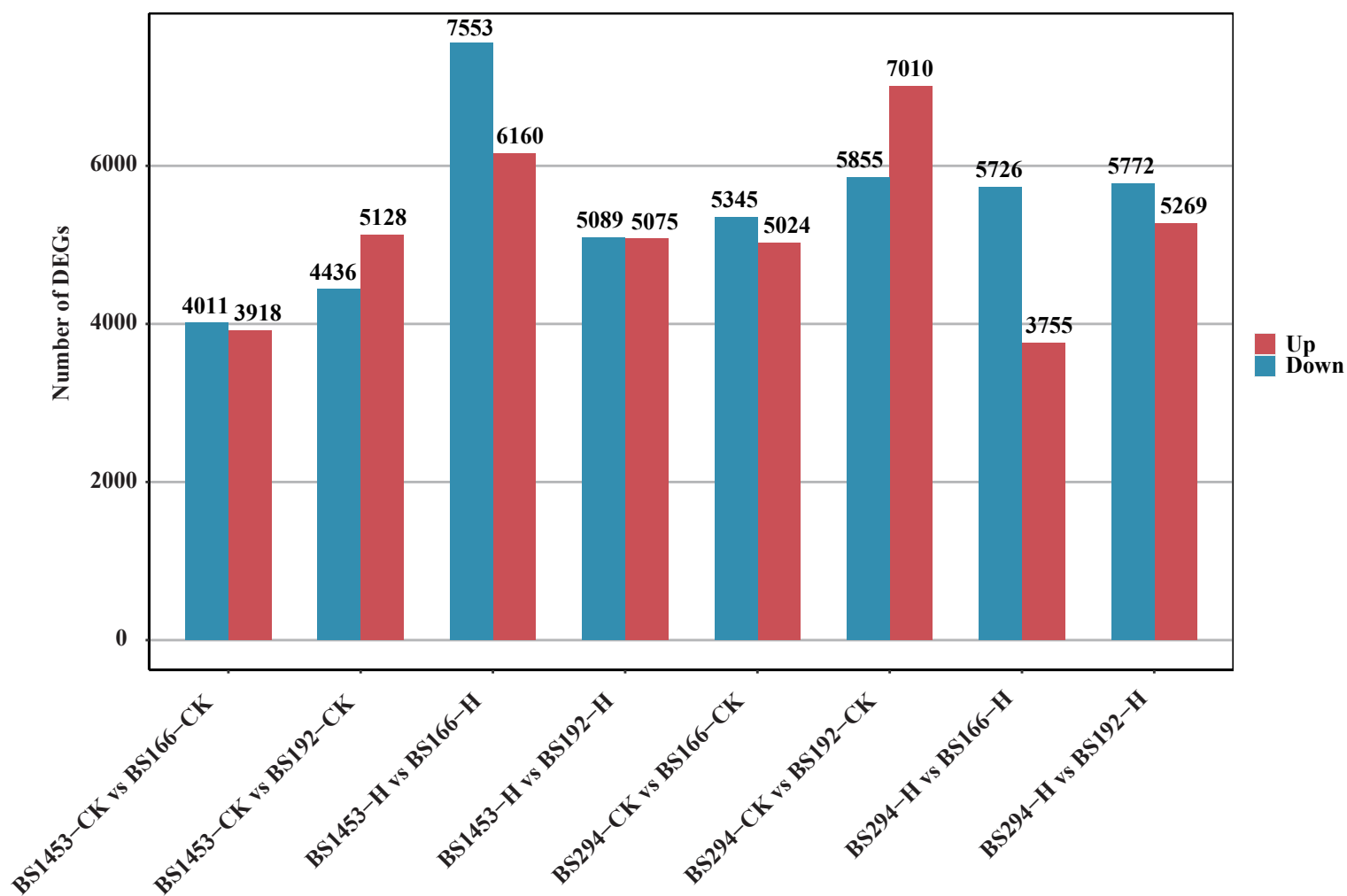

Supplement: Supplementary file 1 [file biomolecules-16-00368-s001.zip › Figure S1.pdf]

A

GO enrichment of Biological Processes

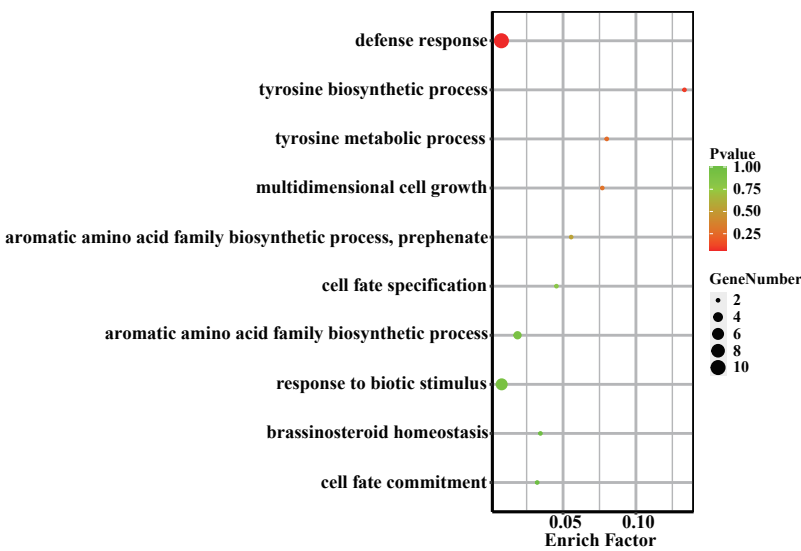

B

KEGG pathways

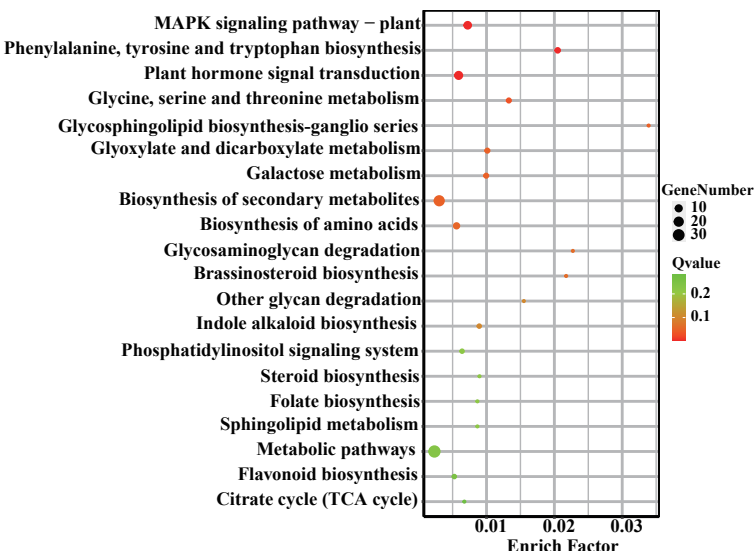

Supplement: Supplementary file 1 [file biomolecules-16-00368-s001.zip › Figure S2.pdf]
